# Effects of Stepwise Lung Recruitment Maneuvers in Patients with Early Acute Respiratory Distress Syndrome: A Prospective, Randomized, Controlled Trial

**DOI:** 10.3390/jcm8020231

**Published:** 2019-02-10

**Authors:** Shu-Chen Kung, Yi-Li Hung, Wan-Ling Chen, Ching-Min Wang, Hui-Chun Chang, Wei-Lun Liu

**Affiliations:** 1 Department of Respiratory Therapy, Chi Mei Medical Center, Liouying, Tainan 73657, Taiwan; clh7810@mail.chimei.org.tw (S.-C.K.); wanlin0810@gmail.com (W.-L.C.); smallwa999@gmail.com (H.-C.C.); 2 Department of Pediatrics, Cathay General Hospital, Taipei, Taiwan; b82401103@yahoo.com.tw; 3 School of Medicine, College of Medicine, Fu Jen Catholic University, Xinzhuang Dist., New Taipei City 24205, Taiwan; 4 Department of Internal Medicine, Chi Mei Medical Center, Liouying, Tainan 73657, Taiwan; wangchingmin@yahoo.com.tw; 5 Division of Critical Care Medicine, Department of Emergency and Critical Care Medicine, Fu Jen Catholic University Hospital, Fu Jen Catholic University, New Taipei City 24352, Taiwan

**Keywords:** acute respiratory distress syndrome, stepwise lung recruitment maneuver, ventilator-free days, ICU-free days

## Abstract

Since the clinical benefit of lung recruitment maneuvers (LRMs) is still conflicting, we performed this prospective, randomized, controlled study to investigate whether LRMs should be used in the routine management of acute respiratory distress syndrome (ARDS). This trial was conducted in four intensive care units (ICUs) to compare application of a modified stepwise LRMs with solely lung-protective ventilation in patients with moderate to severe ARDS within 72 h from the onset. The primary outcome was 28-day mortality, and the secondary outcomes were ventilator-free days and ICU-free days. We collected data on 120 ARDS patients from 2009 to 2012, and there was no difference in 28-day mortality between the two groups (28.3% vs. 30.0%, *p* = 0.84). However, among survivors, patients in the LRM group had a significant longer median duration of ventilator-free days (18 vs. 13 days; *p* = 0.04) and ICU-free days (16 vs. 11 days; *p* = 0.03) at 28 days than in the control group. The respiratory system compliance was significantly higher in the LRM group from day 1 to day 7. The occurrence rate of barotrauma was similar in both groups. We concluded that LRMs combined with lung-protective ventilation in early ARDS may improve patient outcomes.

## 1. Introduction

Acute respiratory distress syndrome (ARDS) is a hypoxic, non-homogeneous pulmonary disease characterized by focal atelectasis, focal emphysema, and intrapulmonary shunting [1,2]. Mechanical ventilation also causes secondary lung injury such as focal emphysema, pulmonary edema, and fibrosis [1,3]. Meanwhile, small tidal volume ventilation can decrease lung damage caused by shearing forces and benefit ARDS patients in terms of mortality and ventilator-free days [4]. This strategy has become the standard lung-protective ventilation strategy in ARDS. Theoretically, pulmonary atelectasis is the major pathological change in ARDS, and an experimental study has shown that tidal ventilation at low airway pressures can augment lung injury [5]. Thus, lung recruitment may improve hypoxemia by opening the collapsed lung and decreasing the intrapulmonary shunt (ventilation/perfusion mismatch) [1,6].

Recently, lung recruitment maneuvers (LRMs) have been shown to safely recruit lung volume and improve blood oxygenation [7,8,9,10]. However, the recruitment cannot sustain the improvement of the clinical outcome of ARDS, and the application of high airway pressure could over-distend the aerated alveoli, leading to secondary ventilator-induced lung injury [11,12]. A stepwise LRM with incremental positive end expiratory pressure (PEEP) and a stable driving pressure can recruit most of the collapsed lung while minimizing hemodynamic compromise and inflammation [8,13]. However, after recruitment, a higher PEEP level could affect the sustainability of this effect [8,14]. Here, we created a modified stepwise LRM with decremental PEEP combined with lung-protective ventilation for patients with early ARDS. We hypothesized that a modified stepwise LRM with individualized moderate to high PEEP would result in a better clinical outcome compared with an established low-tidal-volume strategy in patients with early ARDS.

## 2. Materials and Methods

This prospective, randomized, controlled study was conducted in four intensive care units (ICUs) of Chi Mei Medical Center, Liouying, Taiwan. Patients were recruited from March 2009 through February 2012. The Institutional Review Board of the hospital approved the trial (IRB No.: CLH-0077), and legal substitute decision makers for each patient provided written informed consent. Trial registration: ClinicalTrials.gov, Identifier: NCT01114009.

### 2.1. Patient Population

Patients admitted to the ICUs who met the criteria for ARDS using the American–European Consensus Conference definition [2], who were on mechanical ventilation for <72 h, and who required a ratio of arterial oxygen tension to inspired oxygen fraction (PaO2/FiO2) of ≤250 during invasive mechanical ventilation were enrolled. Before enrollment, oxygen saturation (SpO2) was established to >88% and tidal volume (VT) to 6–8 mL/kg. PEEP and FiO2 were adjusted based on a standard PEEP protocol [8,15,16]. Arterial blood gas was measured after 30 min on these settings to confirm PaO2/FiO2.

After enrollment, the participants were randomized to the experimental group (stepwise LRMs with lung-protective ventilation) or the control group (lung-protective ventilation only). The randomization sequence was created using SPSS for Windows (version 19.0, Chicago, IL, USA) with a 1:1 allocation using random block sizes of 2 and 4 by an independent doctor. The exclusion criteria were age <18 years, pregnancy, pneumothorax or subcutaneous emphysema or severe chronic respiratory lung disease, long-term ventilator dependency, acute brain injury or intracranial hypertension, neuromuscular disease, acute coronary syndrome or persistent ventricular tachyarrhythmias, and premorbid conditions with an expected 6-month mortality risk exceeding 50%.

### 2.2. Study Protocol

**Figure 1:**
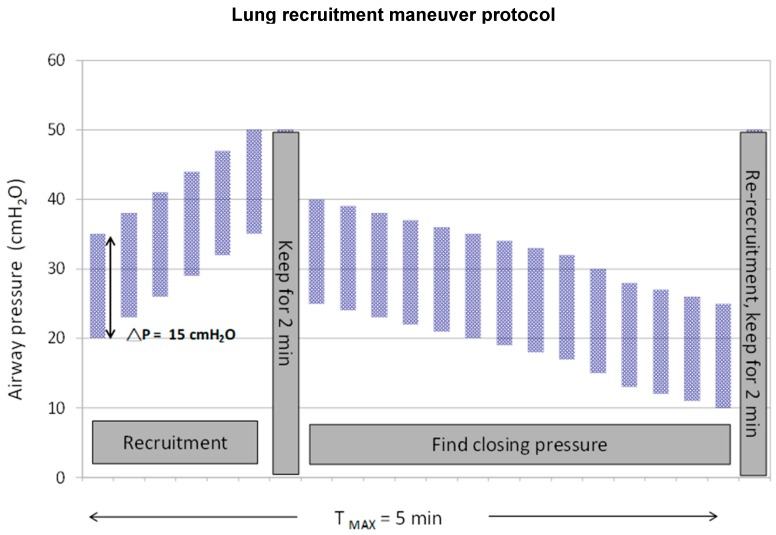
Diagram of a stepwise lung recruitment maneuver followed by decremental positive end-expiratory pressure titration applied in the study. (T _MAX,_ maximum time).

The baseline mechanical ventilation was pressure control ventilation with VT of ≤6–8 mL/kg of ideal body weight and a maintained plateau pressure of ≤30 cmH_2_O (Table S1). For the experimental group, patients were sedated before LRMs and a neuromuscular-blocking agent was administered during LRMs. After ensuring hemodynamic stability, LRMs were performed on pressure control mode and at a peak airway pressure of 35 cmH_2_O, PEEP of 20 cmH_2_O, FiO2 of 1.0, respiratory rate of 15 breaths/min (bpm), and I/E ratio of 1:1. PEEP was increased by 3 cmH_2_O every three breaths up to a peak airway pressure of 50 cmH_2_O, and the ventilation was kept for 2 min. Thereafter, to find a derecruitment point, dynamic compliance (Cdyn) was observed with each breath, as displayed on the SERVO-i ventilator (MAQUET, Rastatt, Germany). We lowered the PEEP to 25 cmH_2_O, then decreased at 1 cmH_2_O every three breaths until maximum Cdyn (derecruitment point, or call closing pressure) was identified. Once the maximum compliance PEEP was identified, PEEP was increased to 35 cmH_2_O to reopen the lung for 2 min, then returned to 2 cmH_2_O above the derecruitment point (Figure 1). If the derecruitment point was not found, PEEP was set based on the original ARDSnet protocol [4]. We adjusted the FiO2 and respiratory rate to maintain adequate SpO2 and minute ventilation, and followed up arterial blood gas 30 min after. LRMs were performed every 8 h until maintaining FiO2 ≤0.40 and PEEP ≤10 for 8 h or more. An additional recruitment maneuver was performed following each disconnect from the ventilator. If PaO2/FiO2 levels were stable or increasing for 12 h or more after LRMs, titrating of PEEP was started with decreases of 2 cmH_2_O every 8 h.

If hypotension (systolic blood pressure <80 mmHg) or bradycardia (<60 bpm) was observed, LRMs were immediately held and a fluid challenge or inotropic agent was administered to maintain haemodynamic stability. The mechanical ventilators used were SERVO-i with Open Lung Tool software, which enables real-time monitoring of respiratory system compliance. SERVO-i continuously displays PEEP, inspired and expired VT, and Cdyn. The graphical display of Cdyn indicates the response of the patient’s respiratory system mechanics to each change in PEEP.

In both groups, the patients were assessed for weaning readiness for a spontaneous breathing trial daily when FiO2 ≤0.4 and PEEP ≤10.

### 2.3. Data Collection and Measurements

Data on demographics, the Acute Physiology and Chronic Health Evaluation (APACHE) II score [17], the multiple-organ dysfunction score [18], and clinical conditions associated with ARDS were collected. We recorded the respiratory system mechanics, gas exchange, and hemodynamic data at baseline and at 8 h intervals thereafter until extubation or day 7 after enrollment. On each day, the lowest and highest values of each parameter were recorded. The patients were followed until day 60 or until discharge while breathing without assistance.

The primary outcomes were 28-day mortality, ventilator-free and ICU-free days at 28 days, and length of ICU and hospital stay after randomization. The secondary outcomes were ventilator-free and ICU-free days among survivors, 60-day mortality, incidence of barotrauma, and transient adverse events during LRMs. Additionally, we compared PEEP, FiO2, plateau pressure, VT, respiratory rate, Cdyn, and gas exchange between groups.

### 2.4. Statistical Analysis

After the first block of 40 patients had been enrolled, a beneficial effect of the LRM approach on ventilator-free days became evident, and we performed an interim analysis. We estimated that a minimal sample of 120 patients was required, with an α of less than 0.05 and a β of greater than 80%. Continuous variables were expressed as mean ± standard deviation with normal distribution or medians (interquartile range, IQR) with non-normal distribution. These variables were compared using the Wilcoxon rank-sum test or Student’s independent *t* test, as appropriate. Categorical variables were compared using the chi-square test or Fisher’s exact test. A multivariate stepwise logistic regression model was used to identify risk factors for mortality.

All analyses followed the intention-to-treat principle, considering all patients in the treatment groups to which they were randomly assigned. In a planned secondary analysis of 28-day mortality, ICU mortality, and hospital mortality, we adjusted for age, APACHE II score, and sepsis. The difference in 60-day mortality between groups was compared using the Kaplan-Meier survival analysis with the log-rank test. All statistical analyses were conducted using SPSS for Windows (version 19.0, Chicago, IL, USA), and *p* <0.05 was considered statistically significant.

## 3. Results

### 3.1. Patients

**Figure 2:**
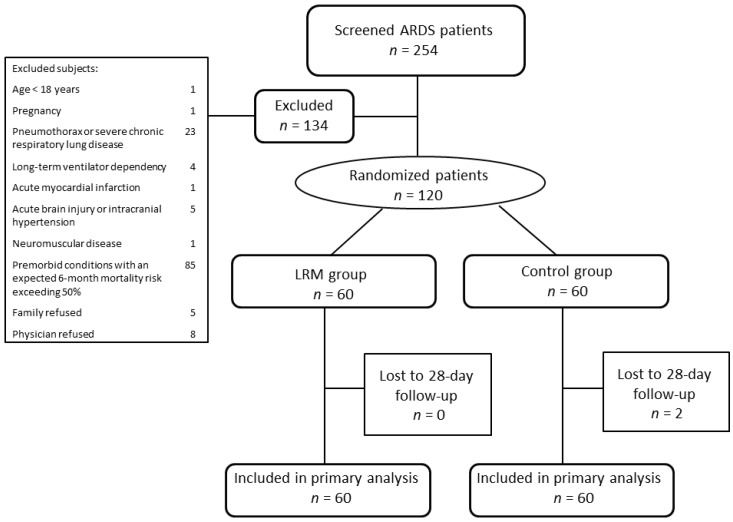
Flow chart of the study. ARDS, acute respiratory distress syndrome, LRM, lung recruitment maneuver.

We screened 254 patients with ARDS; 134 were excluded for reasons listed in Figure 2. The remaining 120 patients were equally randomized to the LRM and control groups. The baseline characteristics were similar between the two groups (Table 1). The most common causes of lung injury were pneumonia (81.7%), non-pulmonary sepsis (10.0%), and acute pancreatitis (3.3%). All patients received sedatives prior to the recruitment maneuvers and 52 patients (86.7%) received neuromuscular-blocking agents in LRM groups compared with 24 patients (40%) in the control group (*p* < 0.001). None had received prone position in either group of patients. Echocardiography was performed in 98 patients (81.7%) and 10 patients (8.3%) had impaired left ventricular contractility, 16 patients (13.3%) had mild to moderate pulmonary hypertension. All patients in the LRM group received at least one recruitment maneuver following the initial recruitment maneuver. The median times of LRMs performed were 5 times (IQR, 4–9 times).

### 3.2. Primary and Secondary Outcome Data

**Figure 3:**
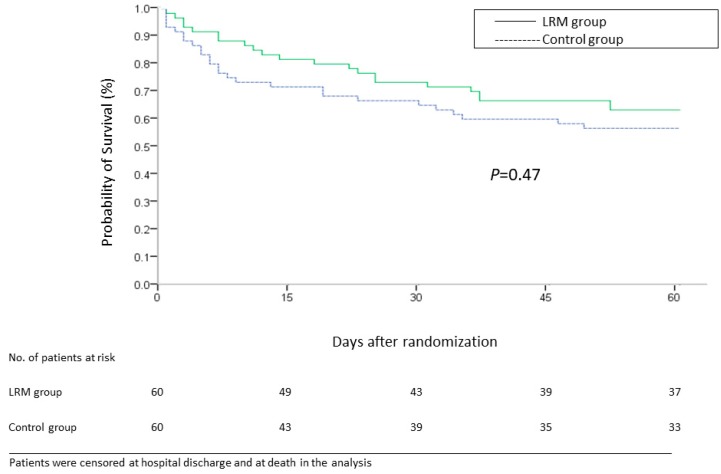
Probability of survival from day of randomization to day 60 among patients in the lung recruitment maneuver (LRM) group and control group.

Two patients were lost to follow up in the control group. No significant difference in 28-day mortality, ICU mortality, and hospital mortality existed between the LRM and control groups (28% vs. 30%, *p* = 0.84, 33.3% vs. 33.3%, *p* = 1.0, 35.0% vs. 38.3%, *p* = 0.71, respectively) (Table 2). The Kaplan–Meier survival curve demonstrates that there was no statistically significant difference in 60-day all-cause mortality between the groups (*p* = 0.47, log-rank test) (Figure 3).

### 3.3. Mechanical Ventilation Days and Other Clinical Outcomes

There were no significant differences in ventilator-free days and ICU-free days at 28 days in both groups (Table 2). On subgroup analysis, when we compared survivors at 28 days who were in LRM group with those in control group, we found that survivors in the LRM group had significantly higher ventilator-free days at 28 days (median = 18 (IQR, 0–21) vs. median = 13 (IQR, 1–18), respectively; *p* = 0.04) and significantly higher ICU-free days at 28 days (median = 16 (IQR, 0–20) vs. median = 11(IQR, 0–16), respectively; *p* = 0.03). The median duration of hospitalization among survivors was 22 days (IQR, 15–34 days) in the LRM group and 34 days (IQR, 20–47 days) in the control group (*p* = 0.07).

### 3.4. Respiratory and Hemodynamic Variables

The mean VT and plateau pressures were similar in both groups on days 1, 3, and 7 and within the target range (Table 3). Cdyn was significantly higher on days 1, 3, and 7 in the LRM group. The closing pressure after recruitment maneuver could be found in 16 patients in the LRM group. The arterial pH, PaO2, and carbon dioxide were similar in both groups at all three times, but the PaO2/FiO2 was significantly higher in the LRM group on days 1 and 3. On day 1, systolic and diastolic blood pressures were also significantly higher in the LRM group, while heart and respiratory rates were similar in both groups on days 1, 3, and 7. 

### 3.5. Adverse Events

Two LRM patients (3.3%) vs. four controls (6.7%) developed a barotrauma episode (*p* = 0.40). In the LRM group, 32 patients (53.3%) experienced transient hypotension, 5 (8.3%) had SpO2 of <88%, 6 (10%) were associated with arrhythmia, and none was associated with cardiac arrest.

## 4. Discussion

This study has several significant findings. To our knowledge, this is the first randomized, controlled trial demonstrating that early application of a stepwise LRM combined with lung-protective ventilation improved patient outcomes, with increased ventilator-free and ICU-free days among survivors. Although no significant difference in 28-day mortality existed between groups, LRMs may have some clinical benefits if applied in the very early phase of ARDS.

Recruitment maneuvers are categorized based on the intervention method. A stepwise LRM with incremental PEEP and a stable driving pressure can achieve most attainable recruitment while minimizing hemodynamic compromise and inflammation [19]. However, this strategy is usually time-consuming and not clinically practical, and the inflation time of LRMs is thought to have less importance in determining recruiting success [20]. Lim et al. performed an extended sigh technique in 20 patients, and an 8 min average was needed [21]. Borges et al. used a stepwise maximum-recruitment strategy that took 20 min for each recruitment maneuver performed [8]. Here, we created a modified stepwise LRM combining these two methods, and only 4–5 min were needed for each maneuver; it was thus less time-consuming. Besides, according to previous studies using a decremental PEEP trial to identify an optimal post-LRM PEEP level that can significantly maintain oxygenation benefits for at least 4–6 h, we added the individualized PEEP levels after LRMs [8,22]. Our modified stepwise LRM protocol is similar to that of Kacmarek et al., which used stepwise incremental PEEP, a stable driving pressure, and post-recruiting decremental PEEP [23]. However, our recruiting peak airway pressure was lower, at 50 cmH_2_O, to prevent over-distension related to hemodynamic adverse events and pneumothorax. Furthermore, the rate of barotrauma (3% vs. 8%), desaturation (8.3% vs. 34%), and arrhythmia (10% vs. 15%) was lower in our LRM group than in Kacmarek et al.’s LRM group [23].

LRMs are crucial in ARDS; however, their impact on the clinical outcomes of ARDS is variable and uncertain. One possible influencing factor of the effectiveness of LRMs is the initial application time. Cavalcanti et al. performed a large multicenter, randomized trial (the Alveolar Recruitment for ARDS Trial, ART) and their findings do not support the routine use of lung recruitment maneuver and PEEP titration in patients with moderate to severe ARDS [24]. For more than 22% patients in that study, however, the duration of ARDS at randomization is above 36 h. In contrast, most of our LRM patients (82%) started LRMs within 24 h after ARDS was diagnosed. Further analysis of our LRM group revealed that patients who received LRMs in very early ARDS (<24 h from ARDS onset) had a significantly lower 28-day mortality, ICU mortality, and hospital mortality, compared with patients who received LRMs within 25–72 h after ARDS onset. Like the beneficial recruiting effect of early prone positioning in ARDS patients, LRMs may better recruit the collapsed alveoli in the early exudative hyaline membrane phase of ARDS than in the late fibroproliferative phase [25,26]. Clinically, Grasso et al. has shown that LRMs successfully improved the oxygenation only in patients with early ARDS who were on the ventilator for <48 h and without impaired chest wall mechanics [26]. Borges et al. used quantitative computed tomography to document that LRMs can reduce alveolar collapse in most patients with early ARDS [8]. Besides, in order to perform LRMs, more patients (86.7%) in our study received neuromuscular-blocking agents in LRM groups compared with patients (40%) in the control group. Papazian et al. found that treatment with the neuromuscular blocking agent in early severe ARDS increased the numbers of ventilator-free days and days outside the ICU [27]. Therefore, LRMs may improve the clinical outcome if applied in very early ARDS.

The etiology of ARDS is another influencing factor of the effectiveness of LRMs. Extra-pulmonary ARDS is believed to be more recruitable [25,28], which can be explained by the higher potential for recruitment in alveolar collapse than in consolidation. Unlike the randomized, controlled trial by Kacmarek et al. and Meade et al., which comprised nearly 50% of patients with extra-pulmonary ARDS, almost 90% of our enrolled patients had pulmonary ARDS in both the LRM and control groups [23,29]. Like two previous studies, our study showed that the LRM group had no significant survival benefit compared with the control group. However, in ARDS survivors, our data showed that LRMs indeed lengthened the ventilator-free and ICU-free days, which could be explained by using LRMs in early ARDS. In pulmonary ARDS, acute lung damage induces interstitial and alveolar edema, followed by rapid fibroblastic cell proliferation and fibrosis [30]. If we perform LRMs in patients with pulmonary ARDS in the very early stage before their pulmonary fibroblastic cell activation, the recruitment effect may become better. However, if the pulmonary damage is so severe that alveolar space is altered by hemorrhage and purulent exudate, the recruitment effect will diminish. Thus, the beneficial effect could be seen in our ARDS survivors. This hypothesis can be proved by Lamy et al.’s morphological study, which showed that PEEP is responsive to patients with less severe lung damage with diffuse congestion and microatelectasis, but this response disappears in late ARDS [31]. Negri et al. also reported an increased collagen content in pulmonary ARDS compared with extra-pulmonary ARDS in the early phase [32]. Therefore, LRMs applied in early ARDS may be more helpful in pulmonary ARDS.

The most common adverse events when performing LRMs are vital sign abnormalities such as hypotension, desaturation, and bradycardia. These hemodynamic adverse events are transient and self-limited, but a small proportion of patients (1%) still need an early cessation of LRMs until they are hemodynamically stable [12]. However, in our study, there was no incidence of LRM termination due to adverse events. There was also low incidence of LRMs associated with pneumothorax, consistent with other previous studies [12,23,33].

Our study has some limitations. First, it was difficult to set the “ideal optimal PEEP” to get the maximum compliance on the deflation limb of the pressure-volume curve of the LRM patients. Optimal PEEP after recruitment could only be found in 25% patients in our study, which may be attributed to the majority of patients who had pulmonary ARDS. The heterogeneous distribution of consolidated/collapsed lung results in a wide variation in pulmonary compliance, making it hard to find the optimal PEEP. Second, our trial was performed in a single institute and the number of cases was limited. Third, only 40% patients received chest CT scan, and the homogeneous of two groups for lung morphological aspects could not be evaluated. Lastly, our primary outcome revealed no difference between the LRM and control groups, and only subgroup analysis showed significant clinical benefits of LRMs. This beneficial impact could have been more notable if our number of cases was larger.

## 5. Conclusions

Applying stepwise LRMs combined with lung-protective ventilation in early ARDS can improve patient outcomes, increasing ventilator-free and ICU-free days among 28-day survivors.

## Figures and Tables

**Table 1:** Baseline characteristics of study subjects.

Characteristics	LRM Group (*n* = 60)	Control Group (*n* = 60)	*p* Value
Age, mean (SD)	66.8 (16.1)	63.7 (20.8)	0.37
Female sex	15 (25.0)	16 (26.7)	0.84
APACHE II score, mean (SD)	20.4 (5.8)	21.5 (6.0)	0.33
MODS, mean (SD)	8.3 (2.5)	9.0 (2.7)	0.18
PaO_2_/FiO_2_, mean (SD)	133.4 (47.0)	129.7 (42.0)	0.66
Tidal volume, mL/kg of ideal body weight, mean (SD)	8.7 (2.1)	8.4 (1.8)	0.37
Minute ventilation, mean (SD)	11.0 (3.9)	10.9 (3.4)	0.86
Total respiratory rate, mean (SD)	22 (6)	23 (7)	0.36
Cause of lung injury			
Pneumonia (%)	52 (86.7)	46 (76.7)	
Non-pulmonary sepsis (%)	4 (6.7)	8 (13.3)	
Multiple transfusion (%)	0 (0)	2 (3.3)	
Acute pancreatitis (%)	2 (3.3)	2 (3.3)	
Others (%)	2 (3.3)	2 (3.3)	

SD standard deviation, APACHE Acute Physiology and Chronic Health Evaluation, MODS multiple organ dysfunction syndrome, PaO_2_ partial pressure of arterial oxygen, FiO_2_ fraction of inspired oxygen.

**Table 2:** Primary and secondary outcomes.

Outcomes	LRM Group	Control Group	Relative Risk (95% Confidence Interval)	*p* Value
	(*n* = 60)	(*n* = 60)		
**Death**				
Death during the first 28 day	17 (28.3%)	18 (30.0%)	0.98 (0.78–1.23)	0.84
ICU mortality	20 (33.3%)	20 (33.3%)	1.00 (0.78–1.29)	1.0
Hospital mortality	21 (35.0%)	23 (38.3%)	0.95 (0.72–1.25)	0.71
**Barotrauma**	2 (3.3%)	4 (6.7%)	0.97 (0.89–1.05)	0.40
**Ventilator-free days ^a,b^**				
Overall population	11 (0–20)	4 (0–16)		0.16
Patients with 28-day survival	18 (0–21)	13 (1–18)		0.04
**ICU-free days ^a,b^**				
Overall population	7 (0–19)	0 (0–15)		0.10
Patients with 28-day survival	16 (0–20)	11 (0–16)		0.03
**Days of mechanical ventilation ^a^**	11 (6–24)	12 (7–23)		0.60
**Days of intensive care**	12 (7–24)	14 (7–27)		0.54
**Days of hospitalization ^a^**	22 (13–34)	23 (10–38)		0.62

LRM lung recruitment maneuver, ICU intensive care unit. ^a^ Continuous data are presented as median (interquartile range). ^b^ Two patients lost to follow-up were excluded for analysis.

**Table 3:** Respiratory and hemodynamic parameters.

		Day 1			Day 3			Day 7	
Variables	LRM Group	Control Group	*p* Value	LRM Group	Control Group	*p* Value	LRM Group	Control Group	*P* Value
No. of patients	60	60		57	55		35	37	
Tidal volume, mean (SD) ^a^,	7.7 (1.0)	7.6 (0.9)	0.57	7.8 (1.2)	7.6 (1.0)	0.49	7.9 (1.3)	7.8 (1.5)	0.62
mL/kg of ideal body weight									
Total respiratory rate, mean (SD) ^a^	21 (4)	22 (5)	0.11	20 (4)	21 (5)	0.14	20 (5)	22 (6)	0.11
Plateau pressure, mean (SD) ^a^	25.3 (3.9)	25.7 (4.6)	0.73	24.0 (4.7)	26.1 (4.3)	0.07	25.2 (5.7)	26.2 (4.4)	0.74
C_dyn_, median (IQR) ^a^	28.2 (25.8–36.3)	25.3 (21.5–28.5)	0.001	31.0 (27.0–36.2)	26.7 (20.9–30.7)	0.001	32 (28.3–38.0)	26.5 (26.0–31.0)	0.002
FiO_2_, median (IQR) ^a^	0.50 (0.47–0.62)	0.55 (0.47–0.72)	0.37	0.38 (0.35–0.46)	0.45 (0.40–0.55)	0.005	0.35 (0.30–0.40)	0.40 (0.35–0.49)	0.04
Set PEEP, mean (SD) ^a^	13 (3)	12 (3)	0.19	11 (4)	11 (3)	0.24	10 (5)	9 (3)	0.47
pH, mean (SD) ^a^	7.40 (0.07)	7.34 (0.33)	0.71	7.43 (0.06)	7.43 (0.06)	0.42	7.46 (0.07)	7.46 (0.06)	0.74
PaCO_2_, mean (SD) ^a^	35.7 (6.1)	36.1 (9.2)	0.70	35.8 (6.3)	37.0 (5.6)	0.23	37.3 (6.7)	36.5 (6.5)	0.85
PaO_2_, mean (SD) ^a^	92.1 (28.2)	84.0 (28.8)	0.06	82.9 (13.9)	79.3 (12.3)	0.19	80.6 (16.5)	80.3 (22.5)	0.40
PaO_2_/FiO_2_, mean (SD) ^a^	174.8 (67.8)	150.8 (62.8)	0.047	209.7 (72.7)	174.1 (55.1)	0.004	222.7 (69.7)	200.3 (71.3)	0.21
Heart rate, mean (SD)	98 (21)	99 (20)	0.65	89 (17)	93 (18)	0.14	94 (20)	96 (17)	0.47
Systemic blood pressure, mean (SD)	133 (20)	119 (20)	<0.001	136 (17)	130 (23)	0.09	137.7 (22.6)	136.8 (28)	0.92
Diastolic blood pressure, mean (SD)	70 (12)	65 (11)	0.03	72 (11)	69 (12)	0.10	74.6 (18.3)	73.0 (14.7)	0.88

SD standard deviation, IQR interquartile range, C_dyn_ dynamic compliance, FiO_2_ fraction of inspired oxygen, PEEP positive end-expiratory pressure, PaO_2_ partial pressure of arterial oxygen, PaCO_2_ partial pressure of arterial carbon dioxide; ^a^ Data shown were derived from the average value obtained for each patient over 3 measurements each day; values were recorded on days 1, 3, and 7 after enrolment.

## Supplementary Materials

The following are available online at https://www.mdpi.com/2077-0383/8/2/231/s1, Table S1: Summary of the ventilator procedure.
