# Supplementary material for: Effects of Stepwise Lung Recruitment Maneuvers in Patients with Early Acute Respiratory Distress Syndrome: A Prospective, Randomized, Controlled Trial
Source: J Clin Med. 2019 Feb 10;8(2):231. doi: 10.3390/jcm8020231 (PMC6406466; doi:10.3390/jcm8020231)
Supplement: Supplementary file 1 [file jcm-08-00231-s001.zip › Table S1--Summary of the ventilator procedure.pdf]

Table S1 Summary of the ventilator procedure

Variables	LRM group	Control group
Ventilator mode	Pressure control	Volume assist-control
Initial tidal volume (mL/kg of predicted body weight)[a]	6-8	6-8
Plateau pressure (cmH_2O)	≤30	≤30
Ventilator rate setting needed to achieve a pH goal of 7.3-7.45 (breaths/min)	10-35	10-35
Ratio of the duration of inspiration to the duration of expiration	1:1 to 1:3	1:1 to 1:3
Oxygenation goal	PaO_2, 55-80 mmHg, or SpO_2, 88-95%	PaO_2, 55-80 mmHg, or SpO_2, 88-95%
Allowable combinations of FiO_2 and PEEP (cmH_2O)[b]	0.3 and 5<br>0.4 and 5-8<br>0.5 and 8-10<br>0.6 and 10<br>0.7 and 10-14<br>0.8 and 14<br>0.9 and 14-18<br>1.0 and 20-24	0.3 and 5<br>0.4 and 5-8<br>0.5 and 8-10<br>0.6 and 10<br>0.7 and 10-14<br>0.8 and 14<br>0.9 and 14-18<br>1.0 and 20-24
Weaning	By pressure support; required by protocol when $FiO_2 \leq 0.4$ and PEEP ≤ 8	By pressure support; required by protocol when $FiO_2 \leq 0.4$ and PEEP ≤ 8

PaO_2 partial pressure of arterial oxygen, *SpO_2* oxyhaemoglobin saturation measured by pulse oximetry, *FiO_2* fraction of inspired oxygen, *PEEP* positive end-expiratory pressure

[a] Subsequent adjustments in tidal volume were made to maintain a plateau pressure of <50 cmH_2O in the group receiving traditional tidal volumes and <30 cmH_2O in the group receiving lower tidal volumes

[b] Further increases in PEEP, up to 34 cmH_2O, were allowed but were not required
